# Site-Specific Glycan Microheterogeneity Evaluation of Aflibercept Fusion Protein by Glycopeptide-Based LC-MSMS Mapping

**DOI:** 10.3390/ijms231911807

**Published:** 2022-10-05

**Authors:** Ju Yeon Lee, Jin-Woong Choi, Seoyoung Hwang, Sung Ho Hahm, Yeong Hee Ahn

**Affiliations:** 1 Research Center for Bioconvergence Analysis, Korea Basic Science Institute, Cheongju 28119, Korea; 2 Graduate School of Analytical Science and Technology, Chungnam National University, Daejeon 34134, Korea; 3 Rophibio Inc., Cheongju 28160, Korea; 4 Department of Biomedical Science, Cheongju University, Cheongju 28160, Korea

**Keywords:** aflibercept fusion protein, glycan microheterogeneity, site-specific evaluation, glycopeptide-based mapping, LC-MSMS, N-glycolylneuraminic acid (NeuGc), I-GPA, q-GPA

## Abstract

The evaluation of the protein glycosylation states of samples of aflibercept obtained from three different regions was conducted by site-specific N-linked glycan microheterogeneity profiling. Glycopeptide-based nano-LC MSMS mapping of tryptic-digested samples of each aflibercept lot provided site-specific information about glycan microheterogeneity on each of the five N-glycosylation sites (two sites in the VEGFR-1 region, two sites in the VEGFR-2 region, and one site in the human IgG Fc region). Next, the glycopeptide-mapping results obtained from the three different aflibercept lots were compared to evaluate the similarity between the samples. Three aflibercept lots showed a high degree of similarity in glycan composition, fucosylation level, sialylation level, and branching, when all five N-glycosylation sites were assessed together as a group. On the other hand, noticeable variations between lots in the glycan types and sialylation levels on the two sites of the VEGFR-2 domain were observed when each of the five N-glycosylation sites were assessed using the glycopeptide-based method. The presence of N-glycolylneuraminic acid (NeuGc) glycans, which may mediate adverse immune reactions in antibody therapeutics, were also detected on the sites of VEGFR1 and VEGFR2 domains, but not on the IgG Fc domain site. These results imply that analyses of the glycosylation profiles of fusion proteins containing multiple N-glycosylation sites, such as aflibercept, being done as a part of quality control for the therapeutics manufacturing process or for biosimilar development, can be done with a more applicable outcome by assessing each site separately.

## 1. Introduction

Protein glycosylation is a post-translational modification (PTM) that can impact a protein’s function or biological activity, such as intracellular transport, cellular localization, secretion, protein-protein interaction, stability, or clearance. Consequently, protein glycosylation can become one of the most important quality attributes related to the development of protein therapeutics, including antibody or Fc-fusion protein therapeutics. Especially, changes in the efficacy or toxicity of protein therapeutics can occur as a result of changes in pharmacokinetics, pharmacodynamics or immunogenicity depending on the nature and extent of protein glycosylation [1,2,3,4]. Biosimilars are defined as biological products that are highly similar to the reference products, notwithstanding minor differences in clinically inactive components. Glycosylation profile is one of the most critical quality attributes (CQA), in terms of the safety and regulatory controls, that is required for biosimilars to closely match the reference products [5].

The majority of protein therapeutics, including aflibercept, are produced from animal cell-based manufacturing systems, after generating stable cell lines using animal cells, such as CHO cells as a host. In contrast to chemical synthesis processes that can be maintained consistently with precision between batches for the manufacturing of small molecule therapeutics, it is practically impossible to maintain identical conditions for the animal cell-based production systems, even when batches were manufactured at the same site with the same procedure. Variations can occur during the upstream bioreactor culturing process, in terms of pH, DO, and temperature, which will cause variations in cell growth, viability and cell density, which will then cause variations in the downstream purification process [6,7,8,9].

Since protein glycosylation is influenced by a variety of elements involved in the production conditions of the manufacturing system, the unique characteristics in glycan microheterogeneity of a therapeutic protein can obviously be varied during the production process. Therefore, a very robust quality control system will have to be applied to make sure that all the upstream and downstream processes are maintained as consistently as possible between batches of production, and to monitor and minimize variations in physicochemical attributes, such as glycosylation profile. The importance of the process and quality controls becomes more obvious for the development of biosimilars. In most cases, the developers of innovative therapeutics will keep their manufacturing process information as proprietary. Therefore, biosimilars developers will have to develop their own process with likely variations from that of the original developers, while maintaining similarity with the reference products with minimum variations in the critical quality attributes [10,11].

Mass spectrometry coupled with liquid chromatography is one of the most powerful tools that can be used to acquire high-throughput and accurate informatics for mapping protein glycosylation patterns [12,13]. Since the protein glycosylation pattern of antibody therapeutics is considered a critical quality attribute affecting a wide spectrum of the biological processes of the therapeutics, precise mapping of glycosylation patterns is very important to evaluate safety and efficacy of the drug [14,15]. Although 2-AB labeling for the glycans, released from IgG monoclonal antibodies, has been a useful glycan mapping method [16,17], this glycan-based mapping shows limitations for the analysis of proteins with multiple glycosylation sites by virtue of the loss of information on site-specific microheterogeneity of a multi-glycosylated proteins, like aflibercept fusion protein.

Aflibercept is a fusion protein between the human vascular endothelial growth factor receptors’ extracellular domains and the Fc portion of human IgG1 [18]. It is comprised of vascular endothelial growth factor receptors 1 and 2 (VEGFR-1, VEGFR-2) fused to the Fc domain of human IgG1, and contains a total of five N-glycosylation sites (two sites from the VEGFR-1 region, two sites from the VEGFR-2 region, and one site from the human IgG Fc region). While most therapeutic antibodies contain a single N-glycosylation site on the Fc portion of the protein, aflibercept, which contains four extra N-glycosylation sites on its VEGF receptor domains, exhibits different glycosylation profiles among glycosylation sites [19]. Consequently, a method of analysis is required that is capable of differentiating the glycan microheterogeneity site-specifically of the multiply glycosylated fusion protein.

A glycopeptide-based mapping method using liquid chromatography-electrospray ionization tandem mass spectrometry (LC-ESI MS/MS) can be a powerful alternative for the glycosite-specific characterization of complex glycoproteins containing multiple N-glycosylation sites [19,20,21,22]. In this study, we used LC-ESI MS/MS for glycopeptide-based mapping of the aflibercept fusion protein. The glycosylation states between the fusion protein lots obtained in different regions were evaluated by site-specifically comparing the glycan microheterogeneity in each N-linked glycosylation site.

## 2. Results and Discussion

### 2.1. Identification of Standard Samples (Korea, Europe and USA)

**Figure 1:**
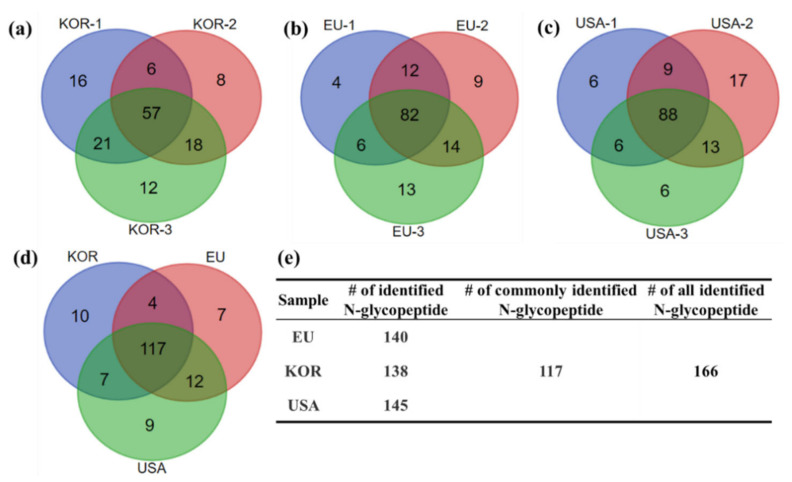
N-glycopeptides identified from I-GPA in (**a**) Korea, (**b**) Europe, (**c**) USA and (**d**) all three standard samples. (**e**) The summary table of the number of identified N-glycopeptides in each sample.

Aflibercept fusion protein (VEGFR-IgG) contains five N-glycosylation sites (two from VEGFR-1, two from VEGFR-2, and one from IgG, as shown in Supplementary Table S1). VEGFR-IgG fusion protein samples from Korea, Europe and USA (STD-KOR, STD-EU and STD-USA) were tryptic-digested and analyzed by LC-MS/MS with three replicates for the identification and quantification of site-specific glycopeptides. The experimental methods in this work were referenced in our previous study, as the reproducibility of replicate sample preparation and LC-MS/MS analyses for glycoproteins were validated [21]. STD-KOR, STD-EU and STD-USA samples were identified 140, 138 and 145 N-glycopeptides, respectively (Supplementary Table S2) from three replicate LC-MS/MS analyses. The number of total identified N-glycopeptides are 166 (Supplementary Table S3 and over 80% (117) of them were commonly identified (Figure 1).

**Figure 2:**
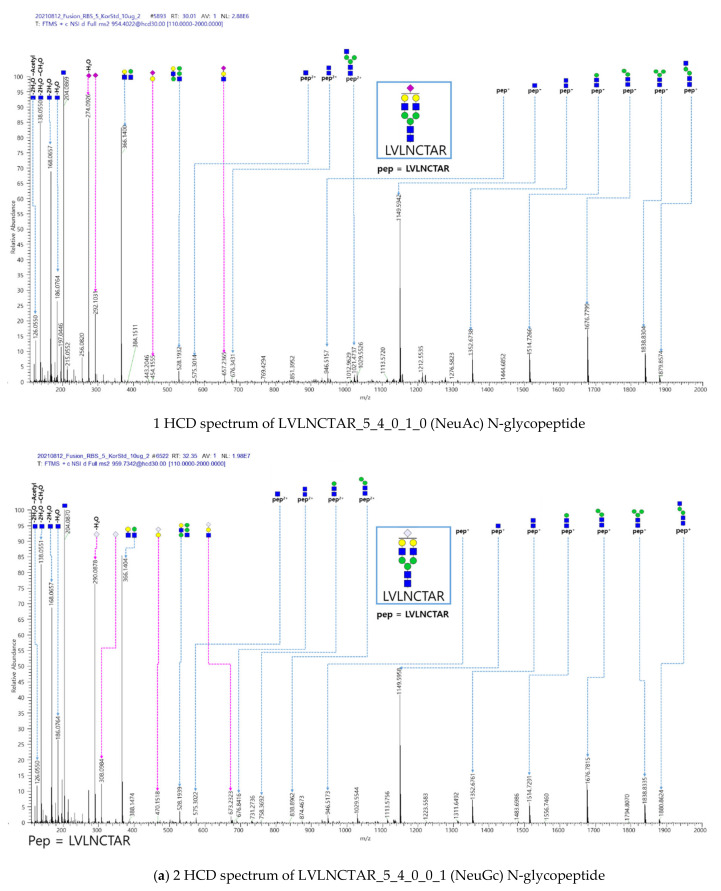

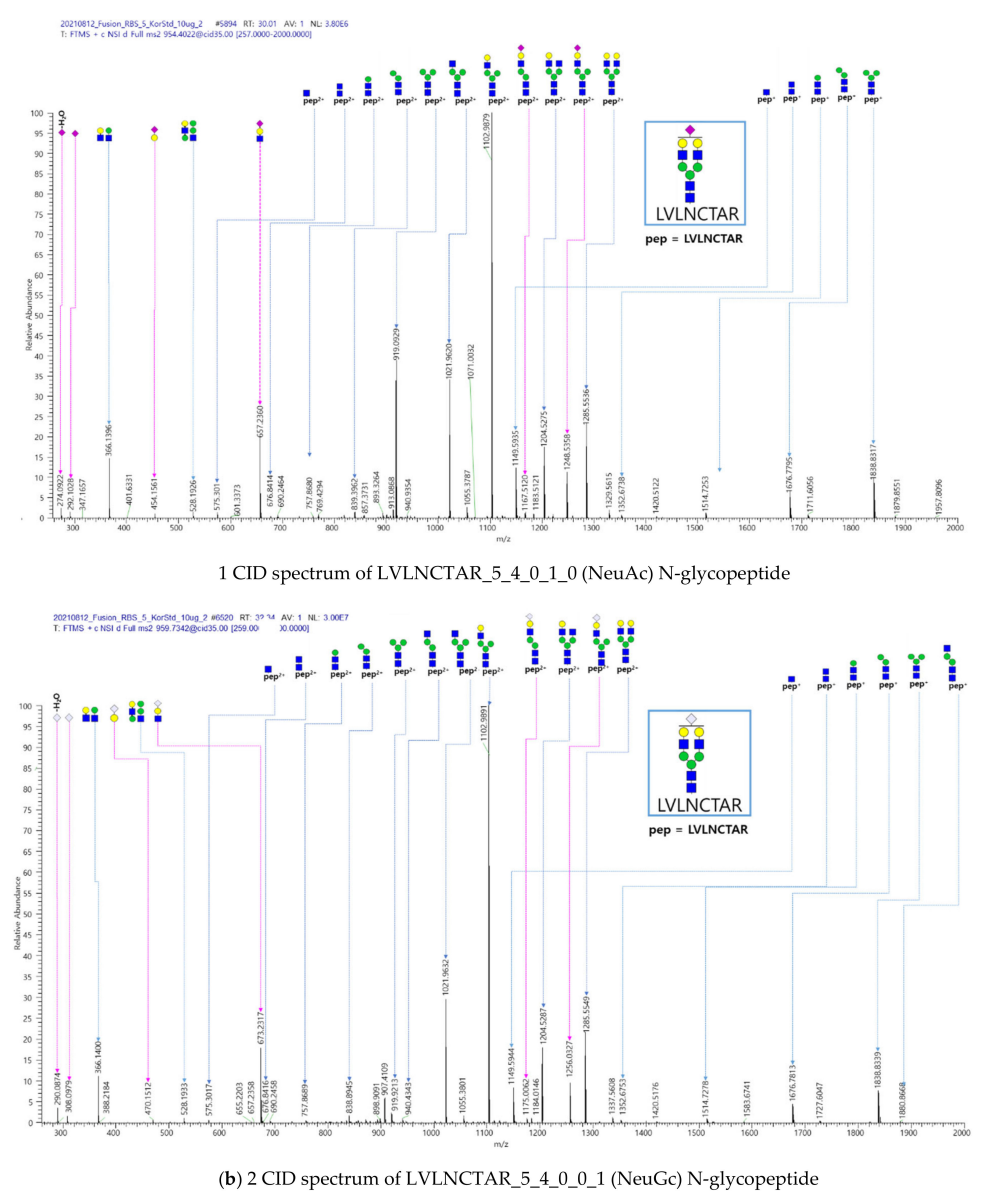

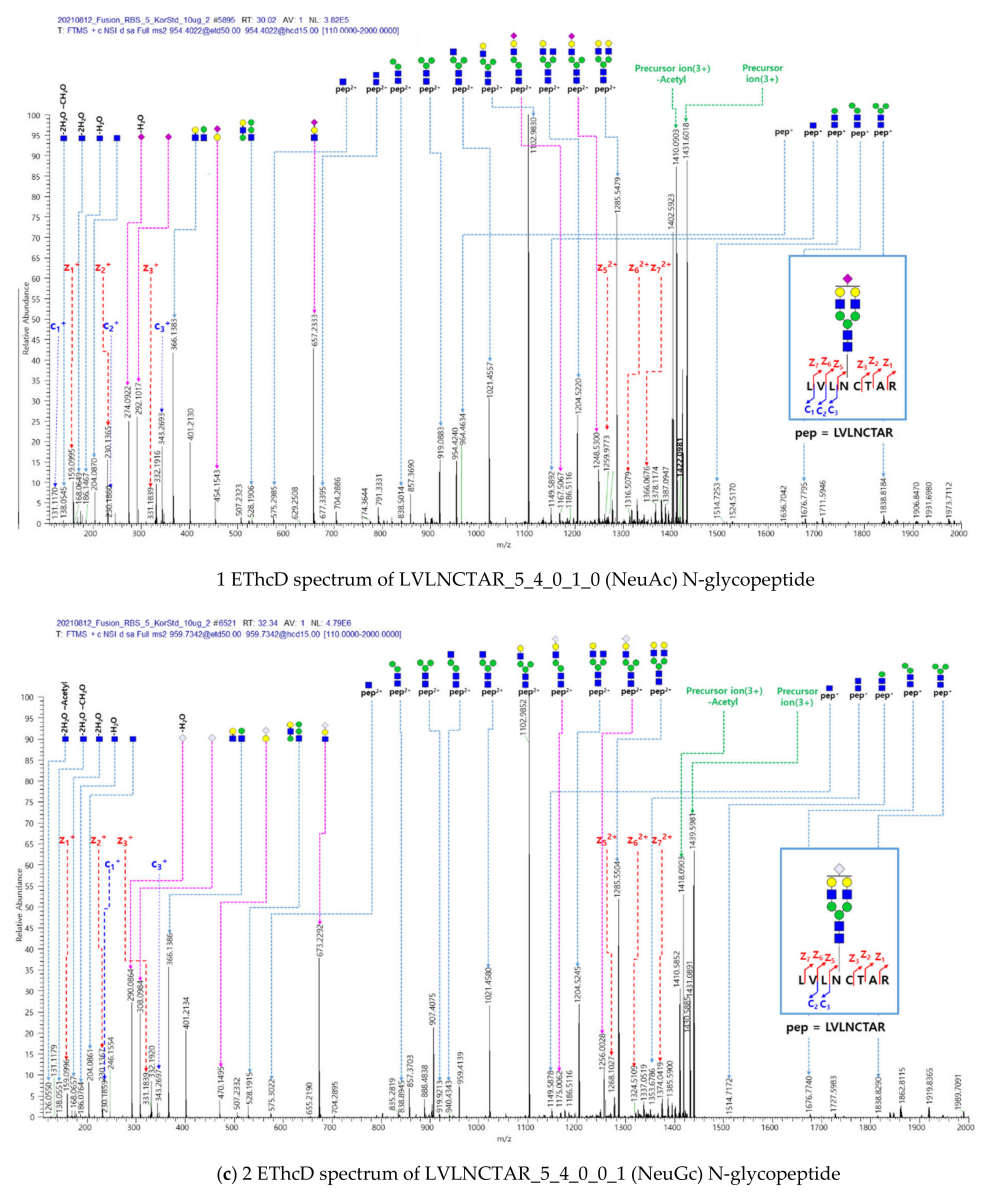
(**a**) HCD, (**b**) CID, and (**c**) EThcD spectra for the LVLNCTAR_5_4_0_1_0 (NeuAc) and LVLNCTAR_5_4_0_0_1 (NeuGc) N-glycopeptides identified from KOR-standard sample by I-GPA search with specific ions (pink dot lines) for NeuAc and NeuGc.

Animal cells, such as Chinese hamster ovary cells (CHO cells), are generally used for producing mAb [23], thus enabling galactose-α-1,3-galactose (α-gal) and N-glycolylneuraminic acid (NeuGc), two non-human glycan structures, to be expressed on mAb [24] at very trace levels. They were negligible because of the anaphylactic responses mediated by anti-α-gal IgE [25] and immune reactions mediated by anti-NeuGc antibodies [26] but were difficult to detect due to their very small level. Here, several N-glycopeptides, including NeuGc glycans identified by I-GPA, were expressed in the VEGFR-1 and -2 domains in three standard samples from Korea, Europe and USA, but not in Fc-IgG domain (Supplementary Table S4). The HCD, CID and EThcD spectra from LVLNCTAR_5_4_0_1_0 (NeuAc) and LVLNCTAR_5_4_0_0_1(NeuGc) identified by I-GPA search are shown in Figure 2 with oxonium ions from HCD, glycan-cleaved glycopeptide fragment ions (B/Y) from HCD and CID, and peptide fragment ions (c/z) from EThcD. The (a)-1, (b)-1, and (c)-1 spectra in Figure 2 contain NeuAc specific oxonium ions (m/z 274.0926 (NeuAc-H2O), 292.1031 (NeuAc), 454.1559 (Hex1NeuAc1), 657.2365 (Hex1HexNAc1NeuAc1)), glycan-cleaved glycopeptide fragment ions (m/z 1167.5120 (Hex3HexNAc3NeuAc1, 2+) and 1248.5358 (Hex4HexNAc3NeuAc1, 2+)) and peptide fragment ions (m/z 1259.9773 (z52+), 1316.5079(z62+) and 1366.0676(z72+) for LVLNCTAR_5_4_0_1_0 (NeuAc)). The (a)-2, (b)-2, and (c)-2 spectra in Figure 2 contains the NeuGc specific oxonium ions (m/z 290.0878 (NeuGc-H2O), 308.0984 (NeuGc), 470.1518 (Hex1NeuGc1), 673.2323 (Hex1HexNAc1NeuGc1)), glycan-cleaved glycopeptide fragment ions (m/z 1175.0062 (Hex3HexNAc3NeuGc1, 2+) and 1256.0327 (Hex4HexNAc3NeuGc1, 2+)) and peptide fragment ions (m/z 1268.1027 (z52+), 1324.5109(z62+) and 1374.0419(z72+)) that were identified for LVLNCTAR_5_4_0_0_1 (NeuGc). The mass difference between NeuAc and NeuGc specific ions is the mass of oxygen.

### 2.2. Quantification of Site-Specific N-Glycopeptides from Standard Samples (Korea, Europe and USA)

**Figure 3:**
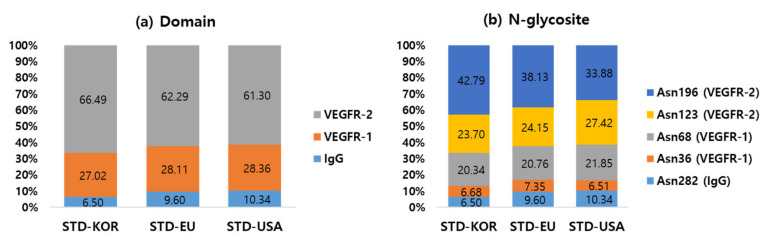
The quantitative distributions were compared according to (**a**) domains and (**b**) site specific N-glycosites among KOR, EU, and USA standards samples.

In total, 166 N-glycopeptides were identified from three LC-MS/MS replications of all three samples using I-GPA (Supplementary Table S3), performed with a q-GPA (quantitated-GPA) [27], for label-free quantitative analysis within the mass tolerance (10 ppm) and the window of retention time (5 min). The quantitative abundance of N-glycopeptides identified at least once in three replicates were extracted from the three replicates data of each sample using q-GPA (Supplementary Figure S1). The intensity of those identified in two or more of the three replicates were averaged and then quantitatively analyzed among samples. Finally, 148, 149, and 148 N-glycopeptides from KOR, EU, and USA standards samples, respectively, were quantified from 155 N-glycopeptides (Supplementary Table S5) and the quantitative results were compared according to the domains (Figure 3a) and the site specific N-glycosites (Figure 3b) in each sample. The abundance of N-glycosylation was approximately 61~66% for VEGFR-2, 27~28% for VEGFR-1, and 7~10% for IgG domain. The site-specific abundance of N-glycosite was arranged Asn196 of VEGFR-2 (34~43%), Asn123 of VEGFR-2 (24~27%), Asn68 of VEGFR-1 (20~22%), Asn36 of VEGFR-1 (7%), and Asn282 of IgG (7~10%).

**Figure 4:**
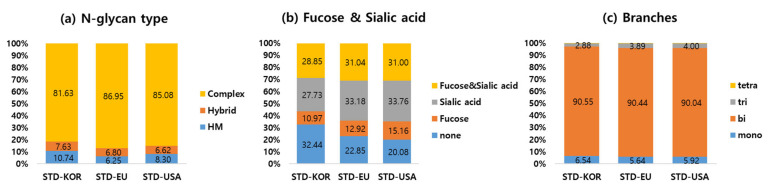
The quantitative distributions were compared according to (**a**) N-glycan type, (**b**) the inclusion of fucose or sialic acid, and (**c**) the number of branches from complex type glycans in KOR, EU, and USA standards samples.

Figure 4 shows the distribution according to (a) N-glycan types, (b) fucose or sialic acid glycans strongly related to the protein stability, and (c) the number of branches of attached glycans in KOR, EU, and USA standard samples. There are three main types of N-glycans: high-mannose (HM), complex, and hybrid glycans. High-mannose is consisted of two HexNAc glycans with many mannoses. Complex contains almost any number of the other glycans (Hexose, Fucose, Sialic acid, HexNAc) with two HexNAc glycans. Hybrid glycans have high mannose on one side of the branch and complex glycans on the other side. The most abundant N-glycan was the complex type, comprising over 80% of those identified in all standard samples (Figure 4a). The percentage of complex type in STD-KOR is less than that of other two samples. The intensity of the complex glycans with fucose & sialic acid is almost similar to that of the complex glycans with only sialic acid. The percentages of fucose and sialic acid, sialic acid, and fucose complex glycans in STD-KOR are less than in other two samples. The percentage of complex type without fucose and sialic acid in STD-KOR was greater than in STD-EU and STD-USA. Complex N-glycan types can also be classified according to the number of branches, such as mono-, bi-, tri-, and tetra-antennary. All standards samples have about 90% bi-antennary complex glycans.

**Figure 5:**
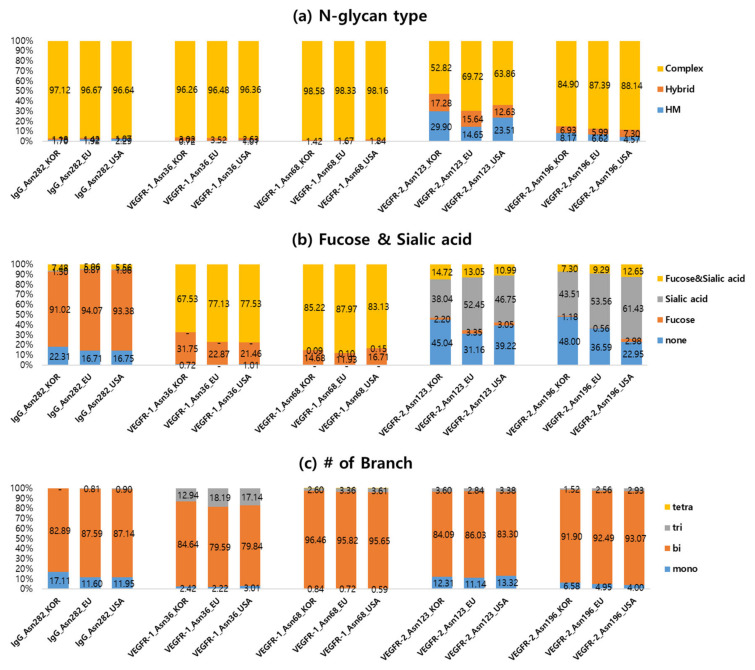
The quantitative distribution for each N-glycosite was compared according to (**a**) N-glycan type, (**b**) the inclusion of fucose or sialic acid, and (**c**) the number of branches from complex type glycans in KOR, EU, and USA standards samples.

Figure 5 shows the distribution of site-specific N-glycosylation according to the type of N-glycan, the complex glycans with fucose and sialic acid, and the number of branches. IgG and VEGFR-1 domains contain over 95% of the complex glycan types, but the VEGFR-2 domain was approximately 53~70% and 85~88% complex type glycans for Asn123 and Asn196, respectively. Asn123 has the most high-mannose type glycans (15~30%) among the five N-glycosites (Figure 5a). IgG dominantly has bi-antennary fucosylated glycans, while VEGFR-1 highly contains bi-antennary fucosylated and sialylated glycans. VEGFR-2 consisted of 38~61% sialylated and 23~45% non-fucosylated/sialylated glycans that were bi-antennary. IgG and VEGFR-2 have about 4~17% mono-branched glycans, while VEGFR-1 has approximately 3~18% tri- or tetra-branched glycans.

**Figure 6:**
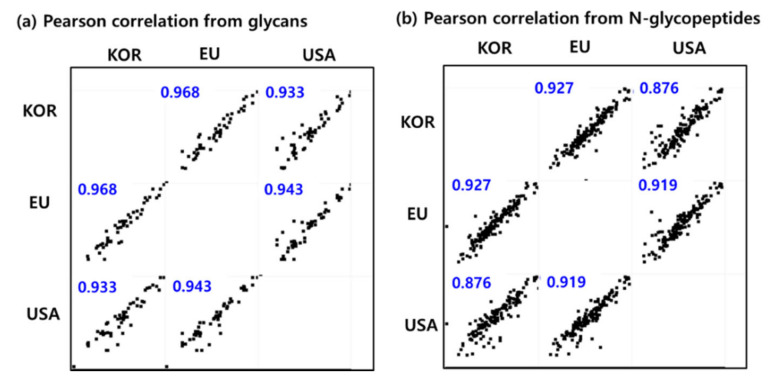
The Pearson correlations from (**a**) 54 glycans and (**b**) 155 N-glycopeptides between three samples.

Finally, the similarity among the three standard samples was represented according to quantitative glycans and glycopeptides with their Pearson correlation values (Figure 6). Figure 6a,b represent the Pearson correlations from 54 glycans (Supplementary Table S6) and 155 N-glycopeptides (Supplementary Table S3), respectively, between the three samples. The similarity between KOR and EU samples, between KOR and USA samples, and between EU and USA samples were 0.968, 0.933, and 0.943, respectively, based on the glycan quantitative analysis, and 0.927, 0.876, and 0.919, respectively, according to the site-specific N-glycopeptide quantitative results. Therefore, the quantification results from the site-specific N-glycopeptides provide us with detailed information regarding the similarity among the fusion protein samples.

## 3. Materials and Methods

### 3.1. Materials

Amicon^®^ Ultra centrifugal filters was purchased from Merck Millipore (Burlington, MA, USA). A C18 trap column from Harvard Apparatus (Holliston, MA, USA) was used. Trypsin from Promega (Madison, WI, USA) was used for protein digestion. Phosphate buffered saline (PBS), 1,4-dithiothreitol (DTT) and iodoacetamide (IAA) from Sigma Aldrich (St. Louis, MO, USA) were obtained for reduction and alkylation. Trifluoroacetic acid (TFA), ammonium bicarbonate (ABC), and formic acid (FA) were also purchased from Sigma Aldrich (St. Louis, MO, USA). Water and acetonitrile (ACN) (MS-grade) were purchased from Merck Millipore (Germany)

### 3.2. Aflibercept Fusion Protein Samples

A total of three different lots of Eylea samples were purchased from the US, Korea, and Europe. Each fusion protein sample was desalted and concentrated using Amicon^®^ Ultra centrifugal filters with a PBS buffer.

### 3.3. Digestion of Fusion Protein

Each protein sample (one hundred μg) with 50 mM ABC was reduced by 10 mM DTT at 60 °C with 850 rpm for 1 hr, alkylated with 25 mM IAA in a darkroom for 1 hr, and digested with trypsin (0.125 μg/ μL, protein:enzyme = 20:1 (wt/wt)) at 37 °C for 16 hr. The digests were dried by vacuum centrifugation and stored at −20 °C until LC-MS/MS analysis was performed.

### 3.4. LC-MS/MS Analysis

The digested sample was reconstituted in DW with 0.1% FA and analyzed by an LC-MS/MS system equipped with an Easy nLC 1200 (Thermo Fisher Scientific, USA) and Orbitrap Fusion Lumos mass spectrometer (Thermo Fisher Scientific) consisting of a nano-electrospray ion (ESI) source (EASY-Spray Sources, Thermo Fisher Scientific). For trapping and separating peptides, a 75 μm × 2 cm C_18_ Pre-column (nanoViper, Acclaim PepMap^TM^100, Thermo Fisher Scientific) and an analytical C_18_ column (75 μm × 50 cm PepMap^TM^ RSLC, Thermo Fisher Scientific) were used, respectively. Flow rate was set at 250 nL/min. Water with 0.1% FA and with 80% acetonitrile, were the mobile phases A and B, respectively. For the LC gradient, 1.6 % B ramped to 4.8% B for 1 min, increased to 8% B (over 12 min), to 28% B (over 60 min), and to 80% B (over 1 min). 80% B was maintained over 5 min, and decreased to 1.6% B for another 3 min. For re-equilibration of the analytical column, 1.6% B was maintained for 8 min before the next run. Approximately 1900 V was the set voltage for producing an electrospray. During the chromatographic separation, the Orbitrap Fusion Lumos was set in data-dependent mode, MS1 and MS2 automatically switched with 3 s of cycle time. The MS data were set using the following parameters: full scan MS1 range was set from 400 to 2500 m/z and acquired by Orbitrap for a maximum ion injection time of 100 ms at a resolution of 120,000 and an automatic gain control (AGC) with a target value of 1.0 × 10^6^; for MS2 spectra, high energy collision dissociation (HCD) and collision induced dissociation (CID) were performed in the Orbitrap mass analyzer at a resolution of 30,000, each with 35% and 30% normalized collision energy and an AGC target value of 1.0 × 10^5^ with a maximum ion injection time of 150 ms. Previously fragmented ions were excluded for 30 s with 10 ppm mass tolerance. The internal calibration was conducted with the mass peak set at 445.12003 m/z, which released from polysiloxane from the silica capillary of the NanoSprayer [28].

### 3.5. N-Glycopeptide Identification

An Integrated GlycoProteome Analyzer (I-GPA) was used to automatically identify N-glycosite-specific glycopeptides from VEGFR-IgG fusion proteins [27]. In summary, N-glycopeptide tandem spectra were first selected with 15 specific oxonium ions of N-glycans. N-glycopeptide candidates were selected for the similarity between their experimental and theoretical MS isotope patterns of N-glycopeptides in the GPA-DB. This GPA-DB combined possible tryptic peptides having N-glycosites of VEGFR-IgG proteins with 351 N-glycans (331 retrosynthetic glycans [29] and 20 polylactosamine series glycans, such as penta- and hexa- [30]). Finally, N-glycopeptides were identified by their Y-score, which represented the degree of matching of the experimental and theoretical fragment ions. From HCD and CID ms/ms spectra, B/Y ions produced from the glycosidic bond cleavage of N-glycans from N-glycosylation sites (oxonium ions and glycan cleaved glycopeptide fragment ions) and b/y ions from peptide bond cleavage (peptide backbone fragment ions), were considered for Y-scoring. The I-GPA search parameters fixed the carbamidomethyl cysteine modification and the mammalian N-glycan for N-sites with one missed tryptic cleavage for peptides. The mass tolerances for precursor and for HCD fragment ions (CID and ETD) were set at 0.02 and 0.02 (each 0.05) Da, respectively. N-glycopeptides within a 0.01 Y-score of FDR were identified from a single targeted glycoprotein.

The nomenclature of the N-glycopeptides was based on the peptide sequence and numbers of hexoses (Hex), GlcNAc, Fuc, N-acetylneuraminic acid (NeuAc), and N-glycolylneuraminic acid (NeuGc) (#Hex_#HexNac_#Fuc_#NeuAc_#NeuGc). The nomenclature of the N-glycans was based on a retrosynthetic glycans database [20]. For example, EEQY***N***STYR_4_4_1_0_0 N-glycopeptide was composed of EEQY***N***STYR (the peptide sequence), 4 (the number of Hex), 4 (the number of N-GlcNAc), 1 (the number of Fuc), 0 (the number of NeuAc), and 0 (the number of NueGc). The italicized and underlined N (***N***) represents an N-glycosylation site.

### 3.6. Glycopeptide Quantification

For label free quantitative analysis of N-glycopeptides, the q-GPA (quantitated-GPA) calculated by the I-GPA software [27] was used as the sum of the three isotopes of the three most intense MS1s. The quantitative abundances of all N-glycopeptides identified from three LC-MS/MS replicates of STD-KOR, STD-EU, and STD-USA, were extracted by q-GPA based on the mass tolerance (10 ppm) and the window of retention time (5 min) of unidentified precursor ions. The intensity from at least two of the three replicates were averaged and then quantitatively analyzed among samples. Finally, uniquely quantified N-glycopeptides were manually inspected, after the retention time had elapsed, for their isotopic mass distribution pattern.

## 4. Conclusions

Through the high through-put nano-LC MSMS analysis of the tryptic-digested samples of aflibercept therapeutics obtained from three different local sites, the protein glycosylation states between the aflibercept therapeutics were compared based on the site-specific N-linked glycan microheterogeneity mapping. The glycopeptide-based mapping results of the five N-glycosylation sites showed high similarity between three aflibercept samples when the mapping data was compared statistically at glycan level. However, in site-specific comparison at glycopeptide level based on the identified 155 N-glycopeptides, notable variances were observed between the aflibercept samples. These decreased correlations between samples mainly originated from the variations in the glycan type and the sialylation level on the two N-glycosylation sites in the VEGFR-2 domain, whereas the other N-glycosylation sites (two sites in the VEGFR-1 region and one site in the human IgG Fc region) showed high similarity between the three aflibercept samples in their glycan microheterogeneity.

In addition, it should be noted that several N-glycopeptides, including NeuGc glycan, were identified from the glycopeptide-based mapping of the three aflibercept samples. The glycopeptides possessing NeuGc glycans were mainly identified on VEGFR1 and VEGFR2 domains, and not on the IgG Fc domain. Since it is supposed that NeuGc glycans mediate adverse immune reactions in antibody therapeutics, the presence of these is not negligible. These results imply that much attention is required to validate the similarity and guarantee the quality of fusion protein therapeutics, and this study shows that the site-specific glycopeptide-based mapping approach is a powerful tool for evaluating fusion protein therapeutics and in developing its biosimilars.

## Data Availability

All data supporting the findings of this study are available within the paper published online.

## Supplementary Materials

The following supporting information can be downloaded at: https://www.mdpi.com/article/10.3390/ijms231911807/s1. Figure S1. Quantitative analysis of 166 N-glycopeptides identified from KOR, EU and USA standard samples, by using q-GPA method. Table S1. The information of VEGFR-IgG fusion protein. Table S2. Site specific N-glycopeptides identified among three samples. Table S3. Site-specifically identified all N-glycopeptides from three samples. Table S4. NeuGc-N-glycopeptide identified by each sample. Table S5. The 155 site specific N-glycopeptide quantitative results among three samples. Table S6. The 54 glycans quantitative results among three samples.
