# Supplementary material for: Site-Specific Glycan Microheterogeneity Evaluation of Aflibercept Fusion Protein by Glycopeptide-Based LC-MSMS Mapping
Source: Int J Mol Sci. 2022 Oct 5;23(19):11807. doi: 10.3390/ijms231911807 (PMC9569749; doi:10.3390/ijms231911807)
Supplement: Supplementary file 1 [file ijms-23-11807-s001.zip › Supplementary figure S1.pdf]

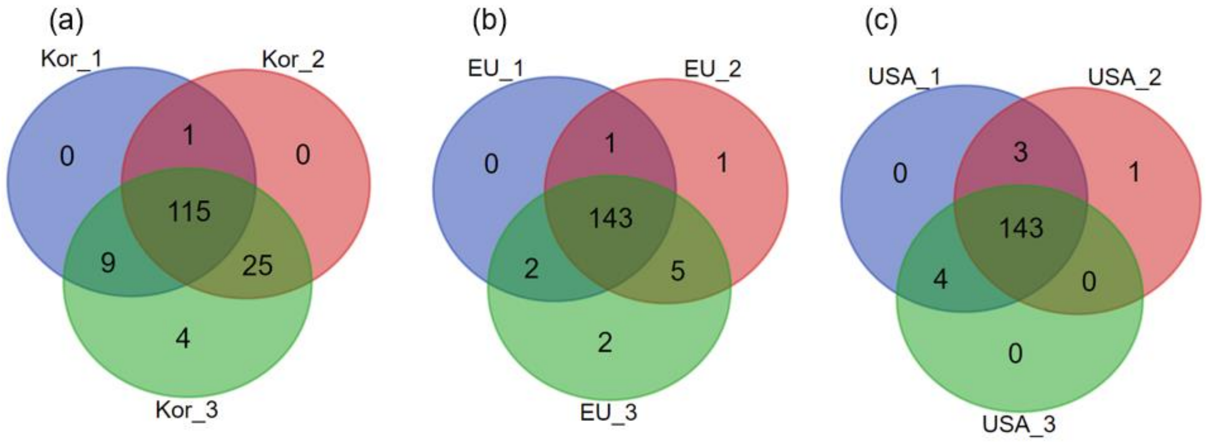

Figure S1. Quantitative analysis of 166 N-glycopeptides identified from KOR, EU and USA standard samples, by using q-GPA method. Total 166 N-glycopeptides identified from three LC-MS/MS replicates of all 3 samples using I-GPA (Supplementary table S3) were performed q-GPA (Reference 27) for label free quantitative analysis within the mass tolerance (10ppm) and the window of a retention time (5min). The number of quantified N-glycopeptides at least two from three replicates by q-GPA were 150, 151 and 150 for KOR, EU and USA standards respectively, as shown in the Venn diagrams.

The intensity from over two from three replicates were averaged and then quantitatively analyzed among samples. Finally, through the manual inspections, 148, 149 and 148 N-glycopeptides from KOR, EU and USA standard samples were quantified respectively and the quantitative results were compared according to domains and site specific N-glycosites in each sample.
